# LabBM Score and Extracranial Score As New Tools for Predicting Survival in Patients with Brain Metastases Treated with Focal Radiotherapy

**DOI:** 10.7759/cureus.7633

**Published:** 2020-04-11

**Authors:** Carsten Nieder, Rosalba Yobuta, Bård Mannsåker

**Affiliations:** 1 Oncology, Nordland Hospital Trust, Bodø, NOR

**Keywords:** palliative radiation therapy, stereotactic radiotherapy, brain metastases, prognostic factors, lactate dehydrogenase

## Abstract

Background

Two recently validated, untraditional prognostic scores include serum albumin and lactate dehydrogenase, among other parameters. The latter are hemoglobin, platelet counts, and C-reactive protein (three-tiered LabBM score), whereas the four-tiered extracranial score includes more than one extracranial site of metastatic involvement. Until now, head-to-head comparisons of these two scores in patients treated with focal radiotherapy for newly diagnosed brain metastases are not available.

Methods

This was a retrospective single-institution analysis of 51 patients, most of whom were managed with first-line stereotactic radiosurgery (SRS). Survival was stratified by the LabBM score and extracranial score.

Results

Both scores predicted survival, but the analyses were hampered by small subgroups. In particular, very few patients belonged to the unfavorable groups. Survival shorter than two months, which was recorded in 14%, was not well predicted by the LabBM score and extracranial score.

Conclusions

Very few patients treated with focal radiotherapy (largely SRS) had unfavorable prognostic features according to the two untraditional scores, which do not include the number of brain metastases and performance status. Additional research is needed to improve the tools that predict short survival because overtreatment during the terminal phase of metastatic disease continues to represent a relevant issue.

## Introduction

Whole-brain radiotherapy (WBRT) was the preferred management option for patients with brain metastases in previous decades [1]. Long-term local control was not particularly relevant because most patients had an active extracranial disease, which inevitably progressed as a consequence of limited systemic treatment options [2]. However, for selected patients with better prognosis and few brain metastases, surgical resection, and later stereotactic radiosurgery (SRS), was offered [3-5]. As a result of better systemic treatment options, prolonged survival, and earlier detection of brain metastases, an increasing number of patients now present in a different situation, i.e., with limited brain disease and need for local control [6-8]. It has been realized that SRS and fractionated stereotactic radiotherapy are technically feasible also in patients with more than three or four brain metastases and that such treatment contributes to increased survival by eliminating the threat of neurologic death [9]. If extracranial disease is absent or controlled, brain control is a prerequisite for sustained survival. Furthermore, preserved quality of life and neurocognition gain importance in patients with survival beyond three to four months [10].

Ideally, patients with limited prognosis would not be exposed to the unnecessary burden of intense treatment, whereas those with better prognosis would receive the therapeutic measures required to prevent neurologic death [11,12]. Several tools (prognostic scores and nomograms with survival and other endpoints) have been developed to support decision-making [13-17]. Initially, they were heavily based on performance status and extracranial disease extent or control, and not stratified by primary cancer type. More recently, specific tumor characteristics have been integrated, e.g., in the molecular lung cancer score, renal cell cancer score, and melanoma score [18-20]. In parallel, it has also been realized that blood biomarkers such as serum lactate dehydrogenase (LDH) and albumin, as well as hemoglobin and C-reactive protein (CRP), may contribute to improved survival prediction models [21]. Berghoff et al. developed and validated a three-tiered score (LabBM), which includes several blood test results (hemoglobin, platelet count, albumin, LDH, CRP) [22]. Median survival in the validation cohort was 10, 6, and 1 month, respectively. Most patients in this study were treated with first-line SRS or surgical resection, i.e., focal brain-directed approaches. Recently, our research group has validated the LabBM score in 167 patients managed with first-line WBRT [23]. Median survival was 4.0, 2.9, and 1.5 months, respectively. The fact that survival was very short in the unfavorable prognostic group is an important advantage over most other prediction models, especially if one intends to reduce treatment-intensity for patients with short survival. If, in contrast, the unfavorable group should contain a subset of long-term survivors, some clinicians would be reluctant to apply the score and argue that undertreatment or withholding radiotherapy would put that subset at a risk of unnecessarily poor survival.

It is still unclear how the LabBM score performs compared with our own four-tiered extracranial score, which includes LDH, albumin, and the number of extracranial organs involved [21]. Identical to the LabBM score, our extracranial score was successfully validated in an independent patient cohort and was the best prognostic model for defining the patients who obviously did not benefit from radiotherapy of brain metastases in terms of overall survival [24]. Unfortunately, the LabBM score had to be excluded because the necessary blood tests were unavailable in this patient cohort. In order to perform the first head-to-head comparison, we tested the LabBM score and the extracranial score in patients managed with first-line focal radiotherapy, an increasingly preferred treatment paradigm.

## Materials and methods

This was a retrospective single-institution study that included all patients with parenchymal brain metastases from histologically verified extracranial primary tumors managed with first-line SRS, SFRT, or other fractionated focal radiotherapy at our hospital. Fractionated radiotherapy prescription was individualized, e.g., 10 fractions of 3.5-4 Gy or 7 fractions of 5 Gy. Further treatment for new or recurrent metastases was individualized too. The strategies consisted of salvage SRS, WBRT, or best supportive care (BSC). Systemic treatment was usually prescribed as judged appropriate by the patients’ medical oncologists and interrupted immediately before and after radiotherapy. Two patients received immune checkpoint inhibitors after radiotherapy (one endocrine treatment for breast cancer, no further systemic treatment for eight patients, and chemotherapy for the remaining patients). The patients were treated between January 1, 2012, and December 31, 2018. Extracranial staging consisted of computed tomography (CT). If clinically relevant, other modalities were added to clarify CT findings, such as isotope bone scan and ultrasound. All blood tests needed to calculate the two scores were routinely assessed approximately one week before radiotherapy (normal values: hemoglobin 11.7-15.3 g/dL for females and 13.4-17.0 g/dL for males; platelets: 130-400 x 109; albumin: 34-45 g/L; LDH < 255 U/l; CRP < 5 mg/L). The extracranial and LabBM scores were calculated as described in the original studies [21,22]. LabBM: 1 point was given for LDH and CRP measurement above the upper limit of normal, and 0.5 points for hemoglobin, platelets, and albumin below the lower limit of normal. A point sum of 0 indicates a favorable prognosis. The maximum point sum was 3.5. Extracranial score: 1 point each was given for elevated LDH, decreased albumin, and more than one extracranial site of metastatic involvement (examples: liver corresponds to one site, whereas liver and bones correspond to more than one site). The sum score ranged from 0 to 3 (3 indicating the worst prognosis).

Overall survival (time to death) from the first day of radiotherapy was calculated using the Kaplan-Meier method, and different groups were compared using the log-rank test (SPSS 25, IBM Corp., Armonk, NY, USA). Only 10 patients were censored after a median follow-up of 14 months (minimum: two months). The date of death was known in all other patients. In addition to univariate tests, the scores were evaluated through a forward conditional Cox regression analysis together with the continuous variables age, number of brain metastases, performance status, and the dichotomized variable extracranial metastases (yes or no). Patients with unknown scores (missing blood test results) were excluded from the Cox regression analysis. Statistical significance was defined as p<0.05 throughout this study.

## Results

The study included 51 patients (27 females, 24 males) largely treated with SRS (n=42). All patients completed their scheduled course of radiotherapy. Further baseline data are shown in Table 1.

**Table 1:** Patient characteristics

Baseline parameter	Number	Percentage
Primary tumor: non-small cell lung cancer	22	43
Breast cancer	6	12
Malignant melanoma	5	10
Renal cell cancer	8	16
Colorectal cancer	8	16
Other primary tumors	2	4
Extracranial status: no extracranial metastases	19	37
Extracranial metastases to one site	15	29
Extracranial metastases to more than one site	17	33
Controlled primary tumor	38	75
Uncontrolled primary tumor	13	25
Number of brain metastases: single brain metastasis	23	45
Two or three brain metastases	17	33
More than three brain metastases	11	22
Salvage treatment: focal brain irradiation	10	20
Whole-brain irradiation	5	10
Median age, range (years)	65, 44-82	
Median Karnofsky performance status, range	80, 60-100	
Median number of brain metastases, range	2, 1-13	
Median time interval (primary tumor to brain metastasis, months)	13, 0-156	
LabBM score: >2 (unfavorable)	2	4
1.5-2.0	6	12
0-1.0 (favorable)	31	61
Unknown	12	24
Extracranial score: 3 (unfavorable)	1	2
2	7	14
1	16	31
0 (favorable)	17	33
Unknown	10	20

According to the LabBM score, most (61%) patients belonged to the group with favorable prognosis. Only 4% were assigned to the group with unfavorable prognosis. In 24%, the score was unknown. With regard to the extracranial score, the following percentages were recorded: 33% favorable, 2% unfavorable, and 20% unknown. As shown in Table 2, patients with a favorable LabBM score had significantly longer survival compared with patients with worse LabBM score (p=0.001).

**Table 2:** Survival outcomes in the prognostic group n.a., not applicable

Score	Median survival in months, 95% confidence interval	Hazard ratio, 95% confidence interval
Extracranial score 3 (unfavorable)	1.7, n.a. (single patient)	n.a., single patient
Extracranial score 2	5.5, 0-13.5	2.8, 0.9-5.0
Extracranial score 1	8.6, 5.0-12.2	1.9, 0.7-3.3
Extracranial score 0 (favorable)	14.6, 10.0-19.2	Reference group
Extracranial score unknown	4.5, 0.3-8.7	
LabBM score > 2 (unfavorable)	1.5, n.a. (two patients)	n.a. (two patients)
LabBM score 1.5-2.0	2.8, 2.0-3.6	5.6, 1.9-9.8
LabBM score 0-1.0 (favorable)	12.1, 9.2-15.0	Reference group
LabBM score unknown	3.8, 1.4-6.2	

**Figure 1:**
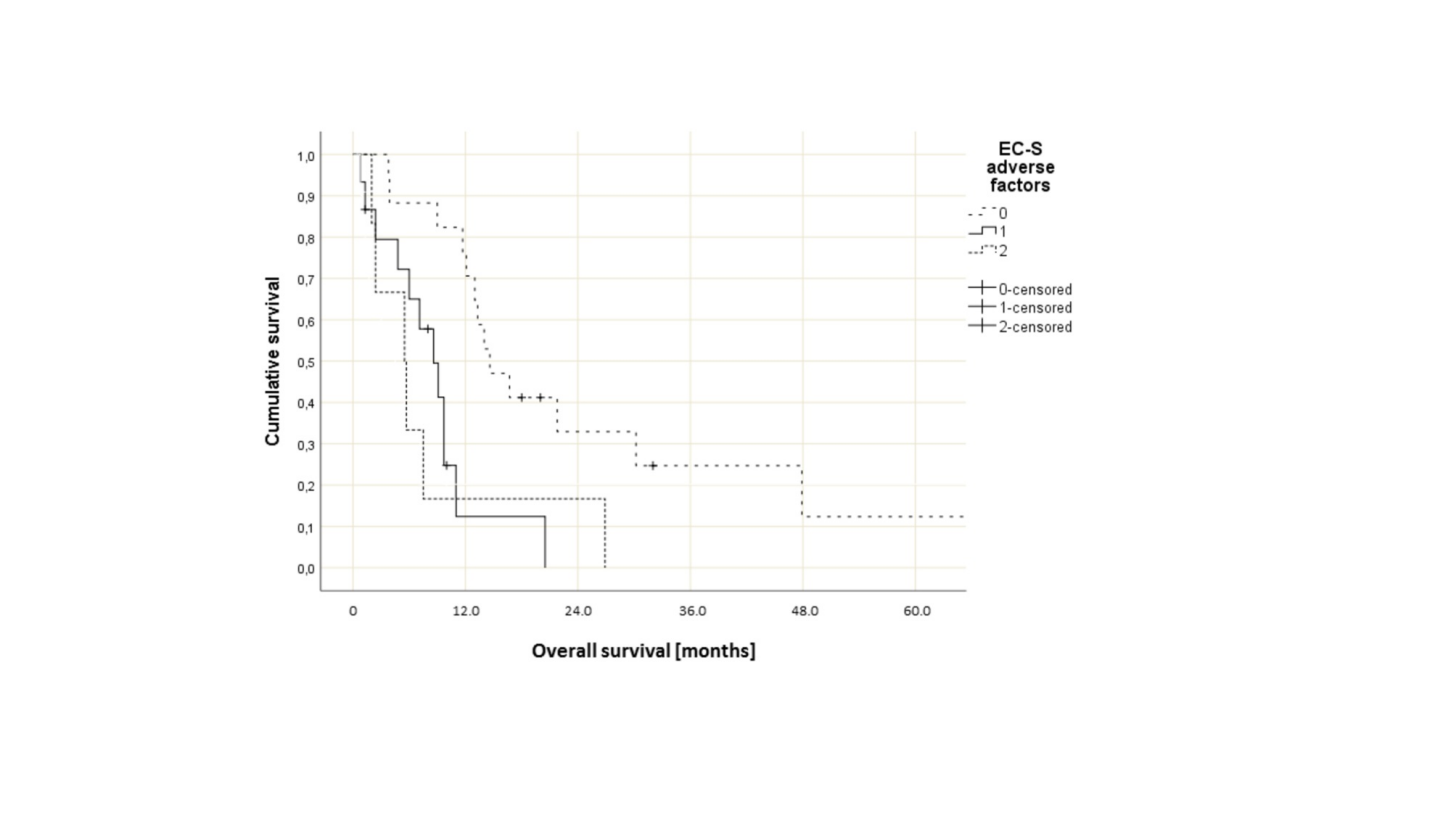
Overall survival (Kaplan-Meier estimate)

However, the latter groups were too small for meaningful statistical analyses. Therefore, the Kaplan-Meier curves are not shown. As also shown in Table 2, patients with favorable extracranial score had significantly longer survival compared with patients with worse extracranial score. The Kaplan-Meier curves displayed in Figure 1 illustrate that the two intermediate groups had largely identical survival.

In Cox regression analysis, the Lab BM score was not significant (p=0.28). The same was true for age, extracranial metastases, and the number of brain metastases (all p>0.1). However, Karnofsky performance status (p=0.001) and extracranial score (p=0.001) were significantly associated with survival. Of the patients, 80% died from uncontrolled extracranial disease and 20% from their brain metastases. Table 3 shows the characteristics of all patients with survival shorter than two months, i.e., those who might not be appropriate candidates for intense local treatment (7/51 [14%]). None of these patients died from neurologic causes.

**Table 3:** Characteristics of all patients who survived for less than two months BM, brain metastasis; DS-GPA, diagnosis-specific graded prognostic assessment; SFRT, stereotactic fractionated radiotherapy; SRS, stereotactic radiosurgery; NSCLC, non-small cell lung cancer

Patient	Therapy	No. of BM	Tumor type	Age (years)	Performance status	Time interval between cancer diagnosis and BMs (months)	DS-GPA	LabBM score	Unfavorable extracranial score	Survival (months)
1	SFRT	3	Colon	69	60	26	0	1.0	No	1.3
2	SFRT	3	Colon	76	60	48	0	Unknown	No	1.0
3	SRS	1	NSCLC	75	70	3	1.5	2.0	No	0.8
4	SRS	1	Urothelial	66	70	21	Unknown	2.0	Yes	1.7
5	SRS	4	Renal cell	72	70	24	1	Unknown	No	1.3
6	SRS	4	NSCLC	81	70	0	1.5	Unknown	No	0.8
7	SRS	2	Rectum	46	70	15	1	2.5	No	1.5

As shown in Table 3, the majority of these patients were not readily identifiable (neither unfavorable extracranial score nor LabBM > 2 points).

## Discussion

This retrospective study was the first head-to-head comparison of the LabBM score and the extracranial score in patients managed with first-line focal radiotherapy, largely administered in the form of SRS. Previously, many patients with less than five brain metastases received WBRT as their initial local treatment [1]. Based on the results of recent studies, an increasing number of patients are currently offered upfront SRS, whereas WBRT is often deferred. For selected cancer types with targetable mutations, upfront drug treatment is sometimes advocated [25]. An important aspect of decision-making is to avoid undertreatment in patients who require effective therapy to prolong their lives and to avoid overtreatment in patients with poor prognostic features. Survival prediction by the use of nomograms and scores is therefore of high clinical relevance, although individual patients may live longer or shorter than predicted by the available models.

In general, predictive models have evolved in recent years. For example, Sperduto et al. have published improved versions of their original diagnosis-specific graded prognostic assessment (DS-GPA) [18-20]. Berghoff et al. used previously underappreciated, inexpensive standard test results to create the now validated LabBM score [22]. As proposed in their publication, the blood test results likely mirror and simplify a variety of other prognostic information such as extracranial disease burden, total tumor burden, cachexia, and systemic inflammatory processes. Comparable with the LabBM score, the extracranial score is also based on other parameters than the traditional ones. Both scores were validated successfully, and the survival of patients with unfavorable prognosis was limited in such a way that the extracranial score may allow for an important aspect in the management of patients with brain metastases, i.e., helping clinicians to make recommendations towards BSC rather than radiotherapy [23,24].

Limitations of this study include the small number of patients, statistical power of subgroup analyses, missing blood test results in approximately 20% of patients, non-standardized imaging of extracranial metastatic sites, and retrospective design. Due to the increased popularity of focal radiotherapy, we limited inclusion to patients treated with this approach and excluded those who had received upfront WBRT. Most likely, this selection process explains the low percentage of patients with unfavorable prognostic scores in our study (<5%). In other words, prescription of focal radiotherapy was obviously limited to patients with better prognostic features, although we did not apply any of the prognostic scores in our routine clinical practice. If we would have applied one of these scores, no blood test results could have been missing.

Probably, the most important finding from this study is the data given in Table 3, which suggests that survival shorter than two months was not well predicted by the LabBM score and extracranial score. This is in contrast to our previous findings in WBRT-treated patients assessed with the extracranial score. In the Cox regression analysis, the LabBM score performed less well than the extracranial score. At present, we are still reluctant to base our clinical recommendations on either score. However, we will continue our efforts toward optimized prognostic models and intend to hybridize the updated DS-GPA variants and the laboratory-based models, hoping that the best of two worlds might hold promise for the future.

## Conclusions

Only a minority of patients treated with focal radiotherapy (largely SRS) had unfavorable prognostic features according to the two untraditional scores, which include LDH and albumin among other factors. Additional research is needed because survival shorter than two months was not well predicted by the LabBM score and extracranial score.
